# Atomistic Insights Into Interaction of Doxorubicin With DNA: From Duplex to Nucleosome

**DOI:** 10.1002/jcc.70035

**Published:** 2025-01-26

**Authors:** Andrea Nedělníková, Petr Stadlbauer, Michal Otyepka, Petra Kührová, Markéta Paloncýová

**Affiliations:** ^1^ Regional Center of Advanced Technologies and Materials, Czech Advanced Technology and Research Institute (CATRIN), Palacký University Olomouc Olomouc Czech Republic; ^2^ Institute of Biophysics of the Czech Academy of Sciences Brno Czech Republic; ^3^ IT4Innovations, VŠB–Technical University of Ostrava Ostrava‐Poruba Czech Republic

**Keywords:** DNA, doxorubicin intercalation, G‐quadruplex, intercalation, MD simulations, nucleosome

## Abstract

Doxorubicin (DOX) is a widely used chemotherapeutic agent known for intercalating into DNA. However, the exact modes of DOX interactions with various DNA structures remain unclear. Using molecular dynamics (MD) simulations, we explored DOX interactions with DNA duplexes (dsDNA), G‐quadruplex, and nucleosome. DOX predominantly stacks on terminal bases of dsDNA and occasionally binds into its minor groove. In the G‐quadruplex, DOX stacks on planar tetrads but does not spontaneously intercalate into these structures. Potential of mean force calculations indicate that while intercalation is the most energetically favorable interaction mode for DOX in dsDNA, the process requires overcoming a significant energy barrier. In contrast, DOX spontaneously intercalates into bent nucleosomal DNA, due to the increased torsional stress. This preferential intercalation of DOX into regions with higher torsional stress provides new insights into its mechanism of action and underscores the importance of DNA tertiary and quaternary structures in therapies utilizing DNA intercalation.

## Graphical abstract

Doxorubicin (DOX) is a known chemotherapeutic drug intercalating into DNA. MD simulations reveal that DOX stacks on terminal dsDNA bases, binds G‐quadruplex tetrads, and intercalates into bent nucleosomal DNA under torsional stress. Energetic analyses show intercalation as the most favorable interaction mode, though requiring an energy barrier in non‐bent DNA structures.

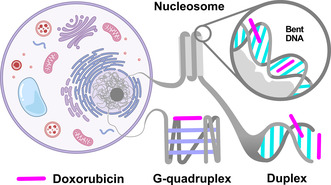

## 1 Introduction

Doxorubicin (DOX) is an anthracycline compound isolated from *Streptomyces peucetius* and widely used as a potent chemotherapeutic agent in the treatment of various cancers, including breast cancer, leukemia, and lymphomas. DOX's mechanism of action is complex and not fully clarified, likely involving a combination of several phenomena. DOX affects multiple cell compartments and functions, leading to cell deaths or impairing cell ability to multiply through various ways [1, 2], including poisoning of topoisomerase II, generating free radicals, affecting sphingolipid metabolism, creating DNA adducts [1, 2], or causing other DNA damage [3]. Anthracycline drugs can even damage chromatin by intercalation into DNA or embedding into minor grooves [3] and destabilizing nucleosomes (NSs) [4]. Despite its efficacy, DOX is associated with significant side effects, particularly cardiotoxicity, which limits its clinical use.

**FIGURE 1:**
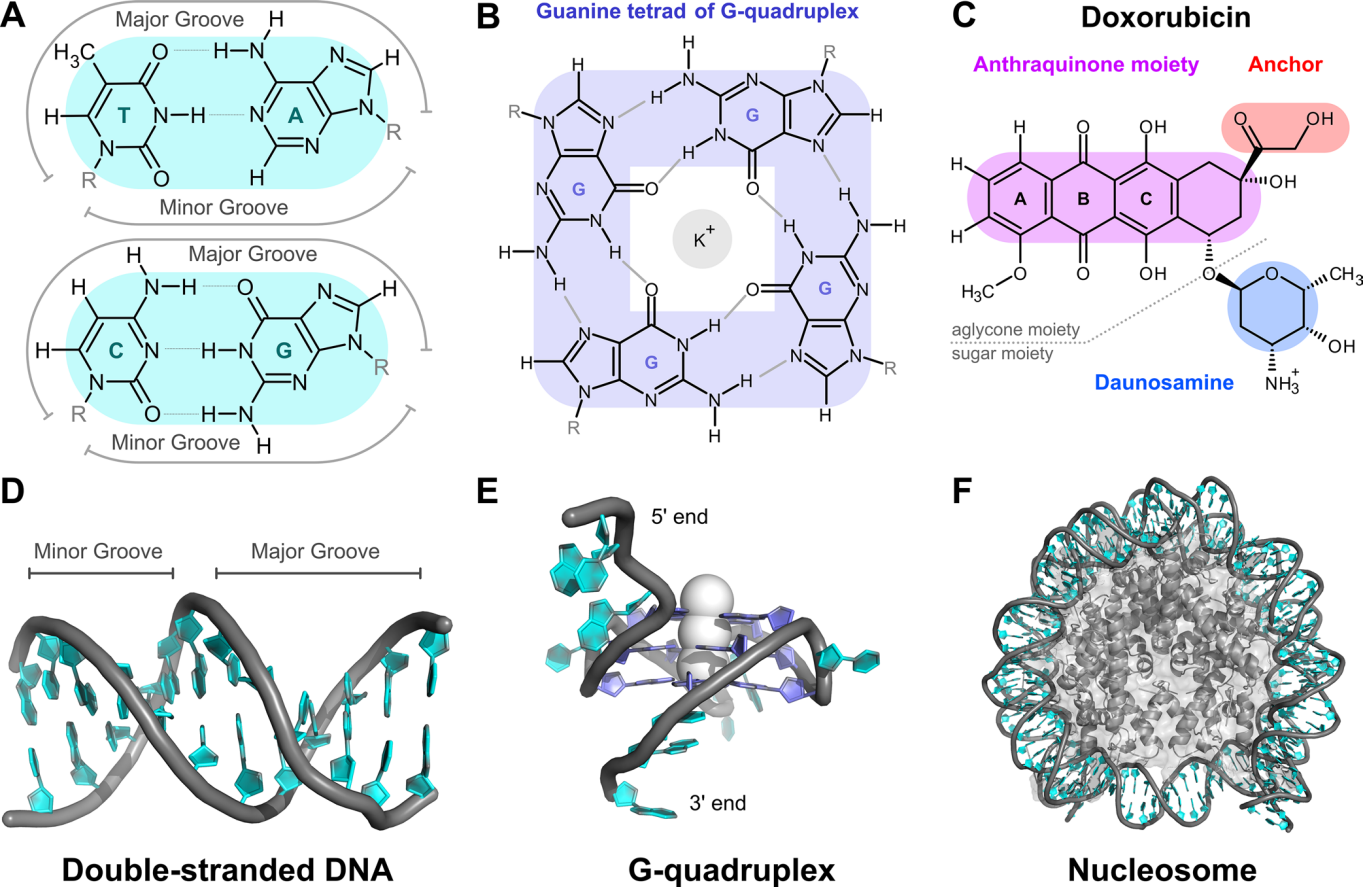
Overview of the studied systems: (A) Canonical DNA base pairs with highlighted major and minor groove sides. (B) Guanine tetrad, a fundamental unit in G‐quadruplex structures. (C) Doxorubicin (DOX) with its functional domains. Rings of the anthraquinone moiety are labeled by letters A, B, and C. (D) Double‐stranded DNA with highlighted major and minor grooves. (E) G‐quadruplex with ions shown within the central channel. (F) Nucleosome structure composed of DNA wrapped around histone proteins, representing the compaction of nuclear DNA.

The chemical nature of DOX and anthracyclines generally offers multiple ways to interact with DNA. DOX consists of a predominantly planar anthraquinone moiety, a sugar moiety known as daunosamine, and an anchor group (Figure 1). The anthraquinone moiety can intercalate between neighboring DNA base pairs [5, 6], blocking further replication and DNA transcription. The positively charged amine group of the daunosamine can enhance interaction with negatively charged DNA backbone or was found to be responsible for histone eviction [7]. Other anthracyclines lacking the sugar moiety exhibit lower anti‐DNA activity and can, e.g., embed in the minor groove [8]. Though several DNA structures with intercalated DOX or similar compounds have been experimentally validated [9], and the intercalation of DOX into DNA is considered as common knowledge, the atomistic details of the intercalation process and its leading factors remain poorly understood.

Molecular modeling can provide very useful insights into biological phenomena at atomistic resolution. Docking studies indicated embedding of DOX into the minor groove [10] or DOX insertion into DNA structures with voids made by manual removal of an intercalated drug [11] or modifications of DNA increasing step distance [12, 13]. Early molecular dynamics (MD) study suggested interactions of DOX with DNA terminal bases and with the major or minor grooves in DNA duplexes [14]. A recent MD study indicated that DOX may also interact with DNA grooves in an NS, but the authors reported a strong tendency of DOX to pack together [15]. To our best knowledge, the only unbiased MD simulation evidencing the intercalation of DOX between nucleobases of DNA duplexes was the pioneering study by Lei et al. [14] The rarely observed intercalation of DOX into DNA structures in MD simulations is somewhat surprising, given the consensus that DOX intercalates into DNA structures [1]. This discrepancy could suggest the presence of barriers for DOX intercalation into DNA structures or highlight gaps in our current understanding of the molecular mechanisms underlying DOX–DNA interactions. Variations in DNA sequences or local environmental conditions, such as ionic strength or pH, might play a role in influencing DOX binding. This complexity extends to the DNA's structural organization, where questions remain about how DNA's secondary and tertiary structures, along with factors such as torsional stress, may affect the mechanism and efficiency of DOX intercalation.

In this study, we performed MD simulations to explore the interaction between DOX and various DNA models, aiming to provide atomistic insights into the process of DOX intercalation and its effects on the DNA structures. Our focus was on DNA structural motifs typically found in chromatin chains, which differ in their organization and mechanical properties. We selected a guanine quadruplex (G4) as a compact, non‐double‐helical DNA model. Guanine tetrads have been predicted to serve as a stacking platform for DOX [16, 17], which may stabilize the G4 structure and inhibit telomerase activity in cancer cells [18]. In contrast, the NS was chosen as a bent, complex structure, characterized by significant torsional stress and representing most of DNA within chromatin. In this structure, DNA is tightly wrapped around a histone protein octamer, creating torsional stress through its interactions with the histones. When a planar molecule such as DOX intercalates between base pair steps, it induces additional torsional stress by unwinding the double helix, potentially destabilizing the NS structure [1]. This increased torsional stress, combined with the unique structural parameters of nucleosomal DNA, makes it a more dynamic and variable target for DOX, unlike the relatively stable, relaxed duplex DNA. The latter was chosen as the third model, serving as a simple, unstressed DNA benchmark.

Through multiple simulations, we observed that DOX predominantly interacted with the terminal bases and the minor groove of the DNA duplex. Furthermore, DOX exhibited a tendency to stack on G4 tetrads, forming additional layers composed solely of DOX molecules. Notably, in our simulations of NS, we identified regions on both sides of the NS dyad that were particularly susceptible to DOX intercalation due to increased torsional stress. We present an atomistic‐level description of the DOX intercalation process, highlighting the stacking of the anthraquinone moiety between base pairs and the interactions of the daunosamine moiety with the negatively charged DNA backbone. Moreover, we identified key DNA parameters influencing DOX intercalation preference, such as elevated fluctuations in the base pair step rise. The insight into the DOX behavior in the DNA regions of high torsional stress may be extrapolated to other high‐stress DNA regions and biological phenomena, such as DNA unwinding. Further, through potential of mean force (PMF) calculation, we identified that though the intercalation of DOX into a relaxed DNA duplex is kinetically hindered by a significant energy barrier, it represents the most thermodynamically favorable interaction mode. Notably, when DOX is intercalated into, e.g., a bent DNA structure, it remains in place even after the release of DNA torsional stress. This knowledge advances our understanding of the DOX mechanism of action in DNA chains.

## 2 Methods

### 2.1 Starting Structures of Biomolecules

The set of studied systems includes three distinct categories of DNA structures representing a broad spectrum of biomolecules. These categories include DNA duplexes, a G‐quadruplex, and an NS. The first category consists of a set of duplexes, including a B‐DNA (PDB ID: 1BNA [19], resolution: 1.9 Å), referred to as dsDNA, which represents a canonical B‐DNA structure, as well as three duplexes derived from NS. These NS duplexes were extracted from different regions during simulations of NS with DOX (see later in the text). The first duplex originates from the region (812:997–921:988) where DOX was intercalated between bases, denoted as I conformation. This duplex was also simulated without intercalated DOX and is referred to as the NS duplex. The second duplex was extracted from region (866:943–878:931) where DOX was embedded in the minor groove, denoted as E conformation. The third duplex was taken from the region (759:1050–768:1041) where DOX molecule was stacked on the terminal base pair, denoted as T conformation. The second category focuses on G‐quadruplex structures, specifically the c‐MYC G‐quadruplex (PDB ID: 6 AU4 [20], resolution: 2.35 Å), denoted as G4. The last category involves an NS structure containing the human H3T histone variant (PDB ID: 3AFA [21], resolution: 2.5 Å), denoted as NS.

### 2.2 Preparation of the Starting Structure of NS and G‐Quadruplex

The starting structure of NS was taken from the aforementioned crystal structure. The protonated states of amino acids were set in agreement with pH 7.3 using the H++ [22]. The system was solvated by combining the OPC explicit water model [23] with excess 0.15 M KCl, using Joung–Cheatham ion parameters [24]. The system was simulated with the AMBER DNA force field OL21 [25], which in addition to the α/γ modification [25] includes the previous [26, 27] *χ*_OL4_, [28] εζ_OL1_ [29], and *β*_OL1_ [30] modifications of the Cornell et al. force field [25, 27] and the ff12SB protein force field [31]. The system was minimized and equilibrated in a manner similar to all systems (see below). The NS structure obtained after 200 ns of MD simulations was taken as the starting point for subsequent MD simulations of NS with DOX. The same protocol, including DNA, ions, and solvent force field parameters, was used for the preparation of G4. Its solvated structure (without pre‐equilibration) was used for further insertion of DOX.

### 2.3 Preparation of the DOX Molecule

DOX topology was prepared following the standard RESP procedure [32] for compatibility with the General Amber Force Field (GAFF) [33]. The molecule was optimized in its protonated state at the B3LYP/cc‐pVDZ level of theory in implicit diethyl ether with Gaussian 16 [34], and the partial charges were calculated at the HF/6–31++G* level of theory, compatible with the AMBER force field family [27]. Atom types and bonded parameters were generated using Antechamber in GAFF, and topology generation was performed by the *tleap* module of AMBER [35].

### 2.4 Building of Systems Containing DOX

Using PyMOL [36], we manually placed DOX molecules to the solvation boxes of the biomolecules as follows: For the dsDNA systems, either one or three DOX molecules were added. Specifically, 30 distinct models were prepared for systems containing one dsDNA molecule (using the same force field parameters as for NS and G4) and one DOX molecule. In 24 of these models, the DOX molecules were placed at various locations along the sides of the solvation box, excluding the terminal base regions. The remaining six models had DOX molecules positioned near both the major and minor grooves, in different orientations (Figure S1). OPC water was added by *gmx solvate* from the GROMACS package [37], and ions were added by *tleap* from the AMBER package [35], which also handled topology generation. For the relaxed NS and G4 systems, 10 and 5 DOXO molecules were added, respectively, to the bulk solvent. Water molecules in proximity to DOX were subsequently removed using VMD [38].

### 2.5 Simulation Protocol

Prior to MD simulations, each system underwent a series of minimizations and equilibrations. Initially, water molecules and ions were relaxed while keeping the positions of the solute constrained allowing the solvent molecules and counterions to move during a 1000‐step minimization. This was followed by a 500‐ps MD simulation under NpT conditions (1 atm, 298 K) to relax the box volume. After this initial procedure, the biomolecule underwent multiple minimization runs, during which decreasing force constants were applied to the sugar‐phosphate backbone atoms. The system was then heated in two steps: First under NVT conditions for 100 ps, followed by equilibration under NpT conditions for an additional 100 ps.

Electrostatic interactions were managed using the particle‐mesh Ewald (PME) method under periodic boundary conditions in the NpT ensemble at 298 K. Temperature control was maintained using the weak‐coupling Berendsen thermostat [39] with a coupling time of 1 ps. The SHAKE algorithm, with a tolerance of 10^−5^ Å, was used to constrain the positions of all hydrogen atoms. A 10.0 Å cutoff was applied to non‐bonding interactions to enable a 2‐fs integration step.

All the biomolecules were simulated individually (i.e., without DOX) as reference systems. To account for variations in DOX positioning, each system was prepared several times with different positions of the DOX molecules in relation to the biomolecule (see Table S1 for details). Simulation lengths varied depending on the system being studied. The NS and dsDNA with three DOX molecules were simulated for 0.75–1 μs, while the G4 systems were simulated for 500 ns. As the simulations of three DOX molecules with dsDNA resulted in DOX stacking of terminal base pair within ~250 ns without further rearrangement, the dsDNA with one DOX molecule was simulated for at least 100 ns per system and until DOX molecule interacted with the dsDNA for at least 20 ns (up to over 800 ns). These simulation lengths were chosen as a balance between computational feasibility and the requirement for sufficient sampling of DOX binding poses. Extending the simulations further was deemed unlikely to produce qualitatively different results. For an overview of all the standard simulations performed, see Tables S1–S3.

### 2.6 Description of Performed Analyses

All the trajectories were inspected visually using VMD and PyMOL. The global behavior of NS and G4 systems was analyzed using the *cpptraj* module (AmberTools) [35]. The program was used for evaluation of the NA structure (root mean squared deviation of atomic positions, dihedral backbone angles, sugar pucker, and helical parameters) and interaction of DOX with NA (monitoring of distances between atoms, radial distribution functions between DOX and residues in contact and number of hydrogen bonds). The most illustrative parameters were rise between the base pairs and twist and are used in the main text and the Supporting Information. Other parameters are not presented due to their redundancy with the analyses already performed. The evolution of these parameters was analyzed throughout the entire simulation, with average values, densities, and other metrics calculated from the last 200 ns of simulation of the complex systems.

Once DOX stacked or intercalated (in)to NA, essentially the only significant movement DOX could undergo was horizontal sliding on the adjacent base pair or G‐tetrad. Therefore, instead of utilizing general‐purpose algorithms such as wavelet analysis [40], we tracked DOX position and orientation relative to the adjacent base pair or G‐tetrad using five collective variables. These included the horizontal displacement of the DOX geometric center (in two dimensions), the rotational orientation of DOX, the preference of a specific orientation at a given point over the base pair or G‐tetrad, and the relative orientation of the DOX daunosamine moiety to the base pair/G‐tetrad along the *z*‐axis.

### 2.7 Calculation of PMF

The thermodynamic evaluation of individual interaction modes was done by calculating PMF profiles. Structures of DOX intercalated between nucleobases (I), embedded in minor groove (E), and stacked on terminal bases (T) were extracted from NS simulations. For each mode, the structures of DOX molecules, interacting DNA bases, and at least five adjacent base pairs in both directions were extracted, resulting in DNA duplexes of 10 base pairs for the I and T conformations and 13 base pairs for the E conformation (for details, see the “Starting Structures of Biomolecules” section). The DNA–DOX structure was then solvated in a rectangular box with 1 nm of solvent in all directions, neutralized by ions, and minimized and thermalized, following the same protocol applied to other systems in this study. Subsequently, 100 ns of unbiased simulations were performed to evaluate the stability of the extracted NS duplex (nucleosomal duplex without the intercalated DOX molecule). The PMF calculations were then performed in GROMACS [37] 2024.1.

A structure obtained from the unbiased simulation was used as the starting configuration for pulling simulations. The parameters were converted to the GROMACS format by *acpype.py* [41], and DOX molecules were pulled away from DNA using an umbrella potential of 2000 kJ mol^−1^ nm^−1^ with a pull rate of 0.1 nm per ns. The biased variable (CV) was defined as the distance between the center of mass (COM) of DOX and COM of interacting DNA bases (terminal base pair for T (759:1050 in NS structure), two base pairs for I (816:993,817:992), and three base pairs for E (871‐873:938‐936)). Frames separated by 0.05 nm in CV were extracted, and umbrella simulations of 50 ns per window were performed with an umbrella potential of 2000 kJ mol^−1^ nm^−1^. Due to persistent interactions between DOX and DNA observed in the furthest frames of the umbrella simulations, we expanded the solvation layer to a cubic box with at least 1.5 nm water layer surrounding the DNA. We then repeated the pulling simulations and extracted additional snapshots for umbrella simulations. Finally, we sampled DOX in bulk water for the furthest frames of all PMFs, which was checked visually. The PMFs were calculated from the final 20 ns of umbrella simulations by the weighted histogram analysis method [42, 43], and the reference value was set to DOX in bulk water. The list of performed PMF simulations is given in Table S4.

## 3 Results and Discussion

### 3.1 DOX Does Not Spontaneously Intercalate Into dsDNA

**FIGURE 2:**
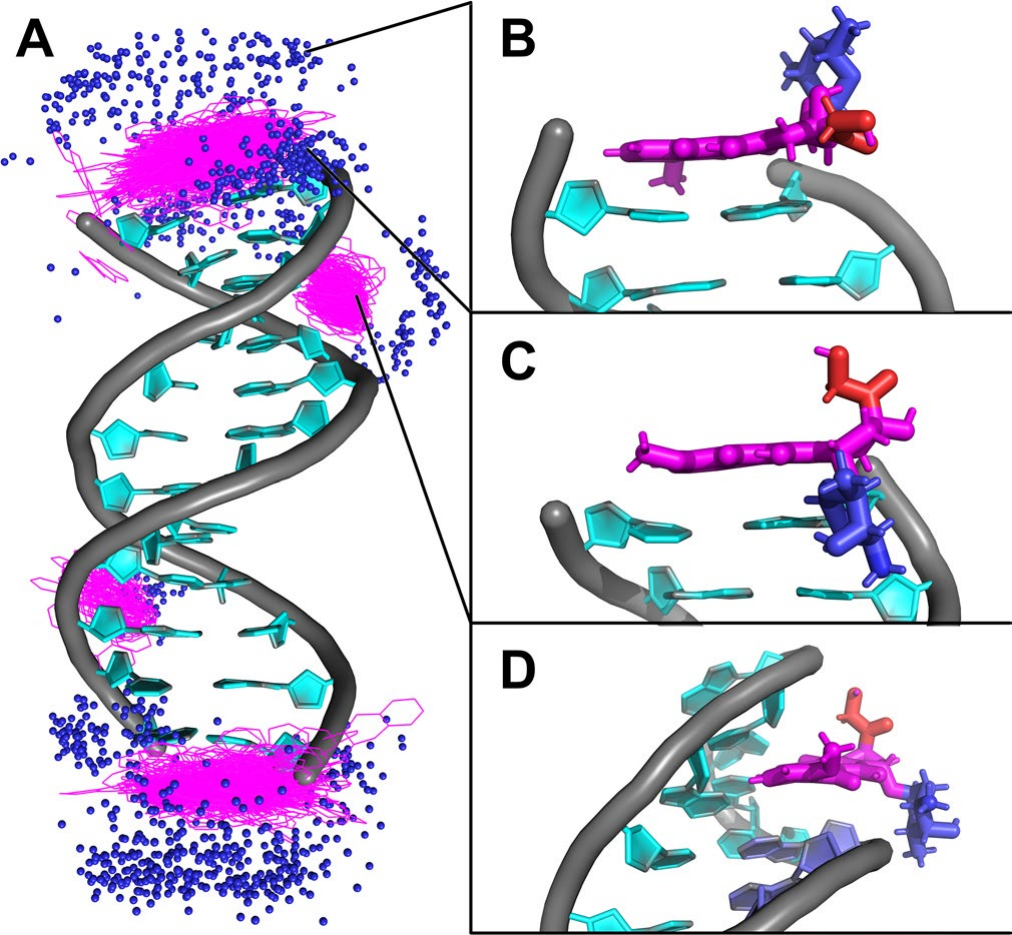
(A) Structure of dsDNA with highlighted positions of doxorubicin (DOX) nitrogen atoms (blue spheres) and the anthraquinone group of DOX (magenta lines) over the final 20 ns of the simulation, superimposed on the starting dsDNA structure. The dsDNA is depicted as a gray‐cyan cartoon, with water and ions omitted for clarity. (B) Atomistic detail of DOX in a stacked conformation, with the anthraquinone moiety shown in magenta, the anchor in red, and the daunosamine moiety in blue, oriented toward the solvent. (C) DOX stacked conformation with the daunosamine facing the major groove. (D) DOX embedded in the minor groove.

To simulate the intercalation process of DOX into a DNA double helix, we prepared 30 independent models of dsDNA each containing a single DOX molecule and performed unbiased MD simulations. In 90% of these simulations, DOX stacked onto terminal base pair (Figure 2B,C), while in 10%, DOX embedded into the minor groove (Figure 2D). For clarity, through the text, we refer to the position of DOX between the nucleobase pairs as “intercalation” and the position of DOX between the DNA backbones in the minor or major grooves as “embedding”. All simulations that resulted in minor groove embedding began with DOX already positioned near the dsDNA. However, in three other simulations where DOX was also placed near the minor or major groove, the molecule instead stacked on the terminal base pair, consistent with the behavior observed in all simulations where DOX started in bulk solvent. Some simulations even interacted with the minor groove but migrated to stack on the terminal bases (Figure S3). The behavior of DOX when stacked on terminal bases varied depending on the initial orientation of the anthraquinone plane on the terminal bases. If the daunosamine group faced the solvent, DOX rotated freely on the terminal bases (Figure S2), only occasionally forming hydrogen bonds between nucleobases and the DOX anchor. Conversely, when the daunosamine group faced one of the DNA grooves, it interacted with the DNA backbone (Figures 2 and S2), occasionally forming hydrogen bonds between the DOX amino group and the backbone phosphate oxygen. In this orientation, DOX showed a preference for interacting with guanine bases in the major groove and cytosine bases in the minor groove (Figure S2). Simulations with three DOX molecules further confirmed the strong preference of DOX for stacking at terminal bases of dsDNA. At both ends, DOX molecules consistently formed stable interactions, including dimers and trimers. In one simulation, a DOX molecule also embedded in the minor groove, maintaining dynamic interactions with the DNA and remaining stably positioned there for over 500 ns. Despite experimental evidence showing that similar anticancer drugs crystallize intercalated within dsDNA [44], our simulations did not observe any spontaneous DOX intercalation between the DNA bases.

Since spontaneous intercalation of DOX into dsDNA was not observed, we focused on its interactions with more complex DNA structures typically found in the cell nucleus, where DOX molecules can be experimentally observed. The G‐quadruplex (G4) presents a large tetrad, which could serve as a preferential target for stacking interactions, while the NS offers multiple binding modes within the double helix wrapped around histone proteins.

### 3.2 DOX Binds to Solvent‐Exposed Tetrads of G4

**FIGURE 3:**
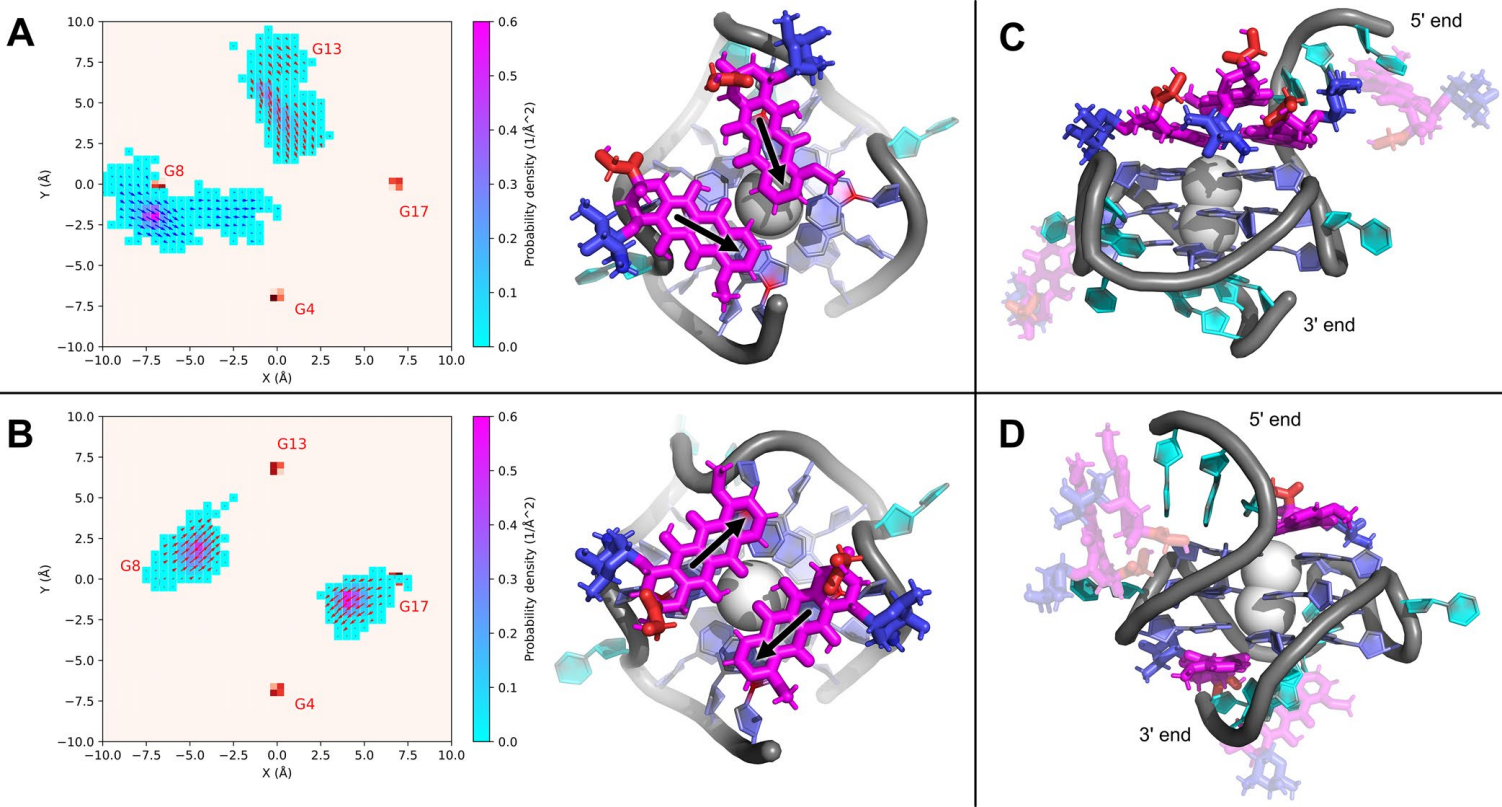
Doxorubicin (DOX) binding to G‐quadruplex (G4): (A) Two DOX molecules bound to the 5′‐tetrad. The plot shows the spatial distribution of DOX above the tetrad, projected into the tetrad plane; for clarity, only the location of the four guanines’ N9 atoms is shown. The position of the geometric center of anthraquinone ring C (cf. Figure 1) is shown by probability density, and orientation of the anthraquinone moiety (from ring C to ring A) is shown by the vector. Its size is proportional to the population density and direction preference at a given location. The red vector indicates daunosamine aiming toward the tetrad, and the blue vector indicates pointing away from the tetrad. The most representative conformer is depicted to the right of the plot, only the key parts of the DOX‐G4 complex are shown for clarity; antraquinone moiety is magenta, daunosamine is blue, anchor is red, stem guanines are slate blue, other nucleotides are cyan, the backbone is gray, and channel cations are white. (B) Two DOX molecules bound to the 5′‐tetrad, but in the opposite fashion compared to that in panel (A). (C) Complex having a DOX molecule stacked onto the platform formed by two DOX molecules bound to the tetrad. (D) Complex having DOX molecules bound to both the 5′‐and 3′‐tetrads.

We performed simulations of G4 with five DOX molecules to monitor their interactions with the G4 structure. Shortly after the simulation started, DOX molecules in the solvent began to multimerize. Binding to G4 happened through the DOX amine group interacting with the G4 backbone or via stacking. DOX molecules could stack on the solvent‐exposed bases of loops, the overhangs or the tetrads, or it could bind in the G4 grooves. DOX molecules could leave these binding sites, except for the one with DOX stacked on the terminal tetrad, which was the most stable. Preferential binding occurred to the 5′‐tetrad, with daunosamine oriented into a G4 groove, where its amino group preferentially interacted with the phosphate group connecting a guanine in the middle tetrad with a guanine in the adjacent tetrad. In fact, two DOX molecules could stack directly on the tetrad plane simultaneously, and their orientations could be either in the same or opposite direction (Figure 3A,B). Additional DOX molecules could stack further on this two‐DOX platform (Figure 3C). Independently on the binding events at the 5′‐tetrad, a DOX molecule could stack on the 3′‐tetrad (Figure 3D). Once accommodated on the tetrad, the DOX molecule(s) position was rather stable with only limited movements (Figures 3 and S4). We believe this rigidity was caused by the interaction between daunosamine and the grooves and/or nearby overhangs. Overhang bases could stack onto DOX from the opposite face than the tetrad to form a sandwiched structure. Neither of the binding modes significantly affected the native G4 stem conformation. Importantly, we did not observe any intercalation of DOX between G4 tetrad planes.

The stacking of DOX on the terminal tetrads of G4 observed in our simulations is consistent with previous theoretical calculations on DOX [16, 45, 46]. This binding mode, including the presence of DOX on both the terminal tetrads concurrently, resembles experimentally observed binding modes of other small molecules to c‐MYC G4 [47, 48, 49, 50, 51, 52, 53, 54]. Yet, the capability of DOX to bind two molecules to the same tetrad simultaneously is rather unique.

### 3.3 Interaction With Nucleosomal DNA

**FIGURE 4:**
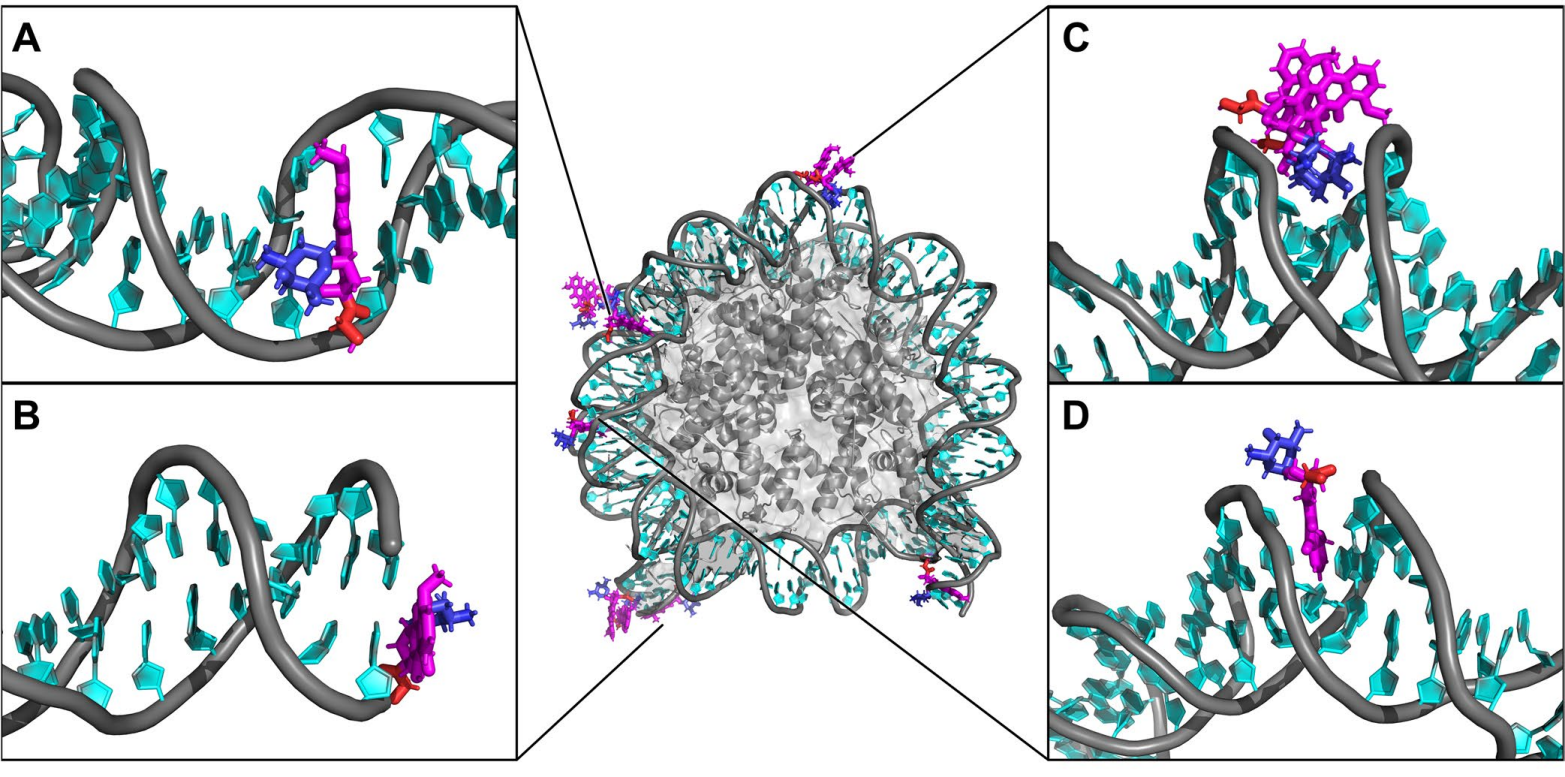
Modes of interaction between doxorubicin (DOX) and the nucleosome (NS): (A) intercalation of DOX between nucleic acid base pairs from the major groove side, (B) stacking of DOX on terminal base pair, (C) embedding of a DOX dimer into the minor groove, and (D) embedding of a DOX monomer into the minor groove. DOX is shown in magenta, blue, and red sticks (see Figure 1), DNA in a cyan/gray cartoon, and the protein in a gray cartoon with a semitransparent surface. Water and ions are omitted for clarity as well as the protein in the insets.

**FIGURE 5:**
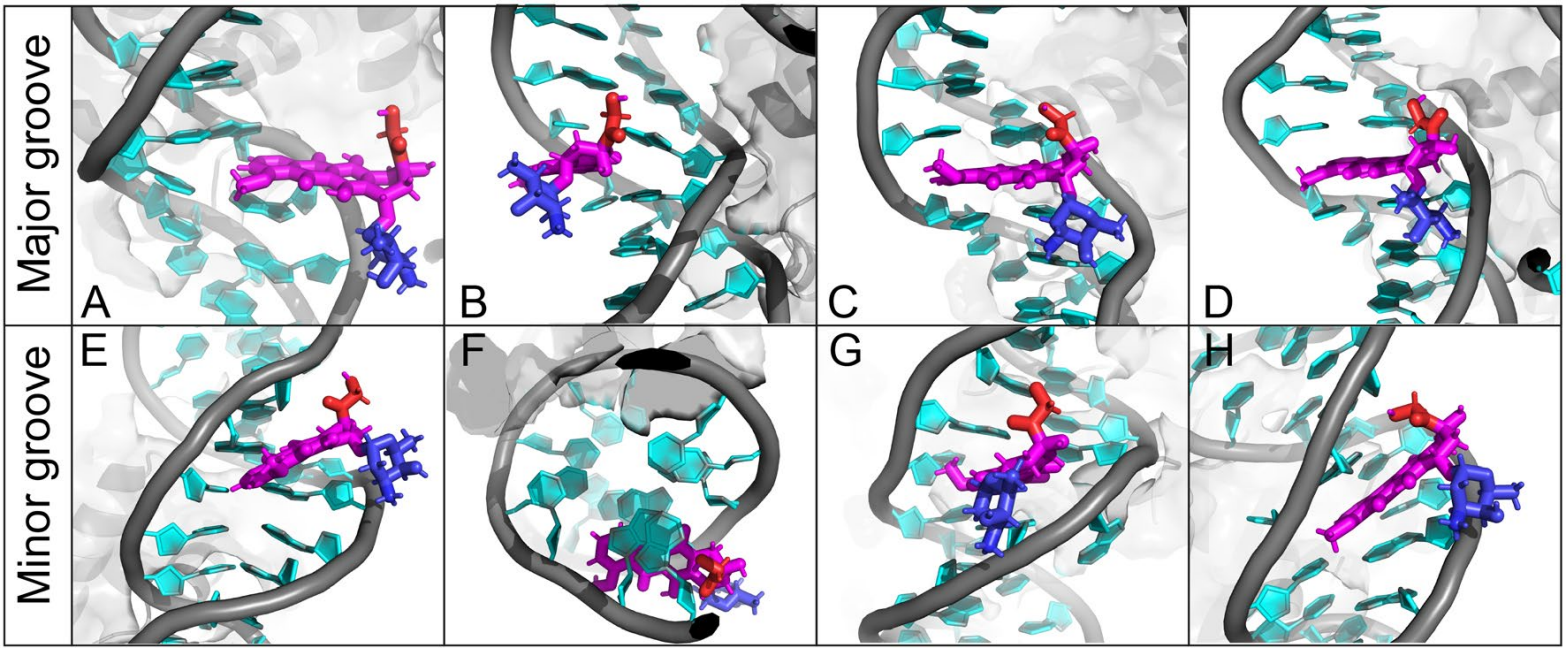
The intercalation process of doxorubicin (DOX) molecule from the major (top panel) and minor (bottom panel) groove sides of the nucleosome (NS). The DOX molecule is shown in magenta/red/blue sticks, while DNA in a cyan/gray cartoon. The NS protein (if visible) is represented as a gray cartoon/surface. Water molecules and ions are omitted for clarity.

The simulations of DOX with NS revealed numerous interaction modes, resulting in a significant variability of DOX interactions. The positively charged DOX molecules, initially placed randomly within the simulation box, were strongly attracted to the negatively charged DNA backbone. As is common for planar molecules [15, 55], some of DOX clustered together, forming multimers (Figure 4), a behavior that has also been observed experimentally [56]. These multimers (dimer, trimer) further enhance the variability of DOX interactions, with some clusters engaging the DNA collectively while others remaining as individual molecules. We observed four ways DOX could interact with NS: (i) stacking on the terminal base pair of NS of individual molecules or DOX clusters (Figure 4B), (ii) intercalation of DOX between base pairs from the major groove side (Figures 4A, 5), (iii) intercalation of DOX between base pairs from the minor groove side (Figure 5), and (iv) embedding into the minor groove, either as single molecules (Figure 4D) or as dimers (Figure 4C). When embedding in the minor groove as a single molecule, DOX molecules positioned their non‐planar functional groups outside of the minor groove, as observed in smaller DNA models [45], and occasionally its anchor or daunosamine moiety hydrogen bonded with the DNA backbone. Embedding in the minor groove has been previously observed in NSs [15], while the intercalation was not observed in previously published simulations of complex stable DNA models.

**FIGURE 6:**
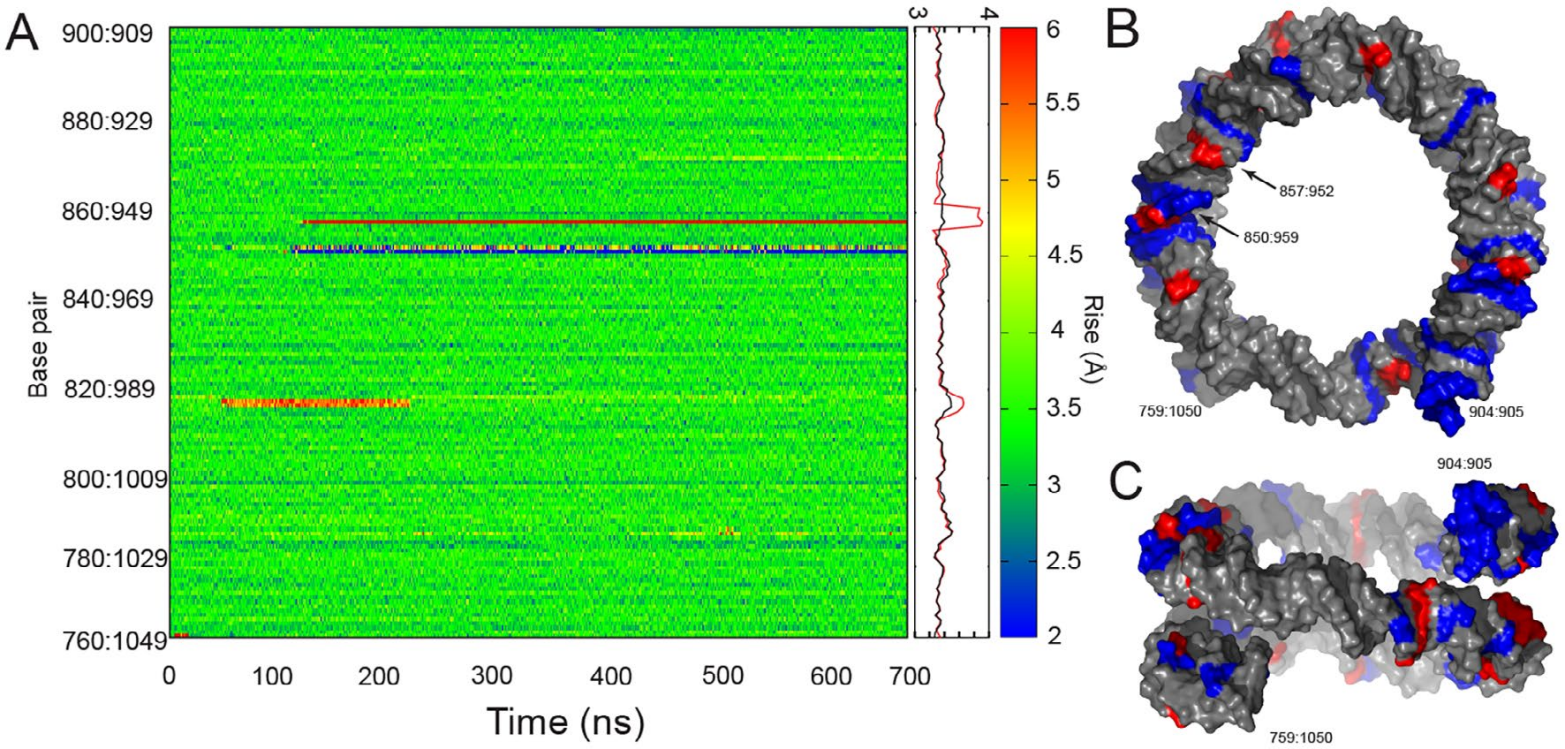
(A) Evolution of the rise between nucleosome (NS) base pairs during the simulation with doxorubicin (DOX, left), along with its running average (middle) over five base pair steps calculated using the sliding window (red curve). For comparison, the running average of the rise parameter from the simulation of bare NS without DOX is shown (black curve). DOX intercalated between base pairs 857:952 and 858:951, and 850:959 and 852:957 (replacing pair 851:958). The region of 816:993 and 817:992 with increased rise is favored for intercalation in another simulation. (B) Surface representation of the NS, colored according to the average rise, highlighting the regions with high (> 3.5 Å, red) and low (< 3.2 Å, blue) rise values, shown from a top view and (C) from a side view.

The intercalation process from the major groove side involved vertical separation of two neighboring base pairs to make space for the DOX molecule. This process was initiated by the interaction of a single DOX in the major groove. The hydrophobic edge of the anthraquinone moiety containing aromatic hydrogens interacted with bases, causing them to increase their roll (Figure 5A) and therefore make more space for DOX. Next, the DOX intercalated between the bases of one strand (Figure 5B), followed by DOX sliding in between the bases of the complementary strand (Figure 5C), further increasing the rise at the affected base pair step (Figure 6). In addition to altering base pair rise, DOX intercalation caused significant changes in the twist of the DNA double helix (Figure S7), introducing additional torsional stress into regions already under strain. The final part of the intercalation process involved optimizing the interactions with the backbone by rotating the flexible functional groups and moving the positively charged amine group close to the negatively charged backbone (Figure 5D). Once intercalated, the DOX molecule stayed between the nucleobases, though its orientation varied. The depth of the intercalation affected the calculated rise parameter, that was the highest with full intercalation (on average 0.52 nm) and smaller (0.48 nm) when DOX was not located between both base pairs (see Figure S5). The most stable DOX was oriented with its anthraquinone almost parallel to the base pairs, while in the pioneering study of small DNA fragments [44], the anthraquinone axis of DOX was more perpendicular to the DNA helix axis.

The intercalation of DOX from the minor groove side was observed only once in our simulations, resulting in a rather shallow intercalation event. The NS sequence contains multiple regions rich in A:T base pairs [57], and in our simulation, DOX interacted within such a region—specifically, a minor groove composed of four consecutive A:T pairs. These base pairs exhibited weak base pairing and a loose stacking pattern, primarily due to the presence of three consecutive TT base steps. The intercalation was initiated with DOX embedding into the minor groove, forming hydrogen bonds with the DNA backbone (Figure 5E). As the buckle parameter of A:T pairs in this region fluctuated, their base pairing became increasingly deformed (Figure 5E). One of the thymine bases moved toward the major groove, likely facilitated by the weak stacking and base pairing in this region (Figure 5F), creating space for DOX to intercalate. Moreover, the intercalation event induced significant conformational changes in the position of neighboring bases. As thymine shifted from its initial position, its base pair partner adenine also began to move, rotating its glycosidic bond from the *anti* to *syn* conformation (Figure 5G). Afterward, adenine reinserted itself into the DNA duplex, though it remained in the *syn* conformation. In response, DOX stacked with an adjacent G while maintaining hydrogen bonds with both strands of the DNA backbone (Figure 5H). Unlike major groove intercalation, this minor groove event did not result in elongation of the DNA strand, as DOX effectively replaced one nucleobase. Despite the shallow intercalation, the interaction remained stable throughout the rest of the simulation, lasting over 0.5 μs.

### 3.4 Prediction of Intercalation Regions

We observed that DOX did not intercalate between base pairs in B‐DNA (see the dsDNA section), the canonical form of DNA characterized by its regular, right‐handed double‐helix structure. The B‐DNA maintained a uniform helical rise (Figure S5) and exhibited considerable rigidity, which may have limited the accessibility of its base pairs for intercalation. In contrast, DOX successfully intercalated into nucleosomal DNA, which is tightly wrapped around eight histone proteins, forming a more complex structure. Nucleosomal DNA deviates significantly from the regular B‐DNA conformation due to its interactions with histones, resulting in altered helical parameters such as twist, tilt, and rise (see Figures S5 and S6 for a comparison of the rise for both DNA structures) [58, 59]. These deviations are symmetrically distributed along the NS DNA chain with the center of symmetry at the dyad [59]. The tight wrapping around histones distorts the major and minor grooves of NS; the major groove becomes narrower and less accessible, while the minor groove often widens and makes direct contact with histones [60]. This wrapping also induces bending, bringing some DNA regions closer together while others are pushed farther apart, affecting interactions with proteins. Additionally, the base pairs are not perpendicular to the helical axis, instead, they are tilted to accommodate the curvature around the histone core.

In our simulations of the NS, we identified regions with altered helical parameters. These regions exhibited changes in twist, which alternated between increased and decreased values (Figure S7) and also the rise of base pair steps was elevated, even in the absence of intercalated DOX (see Figures S5 and S6). Notable, this pattern of elevated rise was observed in both simulations of DOX interacting with different NS regions and in simulations of bare NS. The elevated rise in these regions may serve as potential sites for DOX interactions, as the increased rise could facilitate easier unstacking of base pairs, making these areas more susceptible to intercalation. Experimental observations have confirmed the presence of regions with higher rise in nucleosomal DNA, further supporting the hypothesis that these areas may act as hotspots for drug interactions [61]. Additionally, the observed variations in both twist and rise can be interpreted as indicators of increased torsional stress within specific regions of nucleosomal DNA following DOX intercalation. In our simulations, these regions were symmetrically localized on both sides of the dyad, near the bends in the DNA chain. Interestingly, no strong sequence preference for DOX intercalation was observed; two intercalations occurred between CG base pairs, while another one occurred between a CG and AT pair. Instead, DOX appeared to preferentially target regions with increased torsional stress (Figure 6).

### 3.5 Intercalation Overcomes an Energy Barrier

**FIGURE 7:**
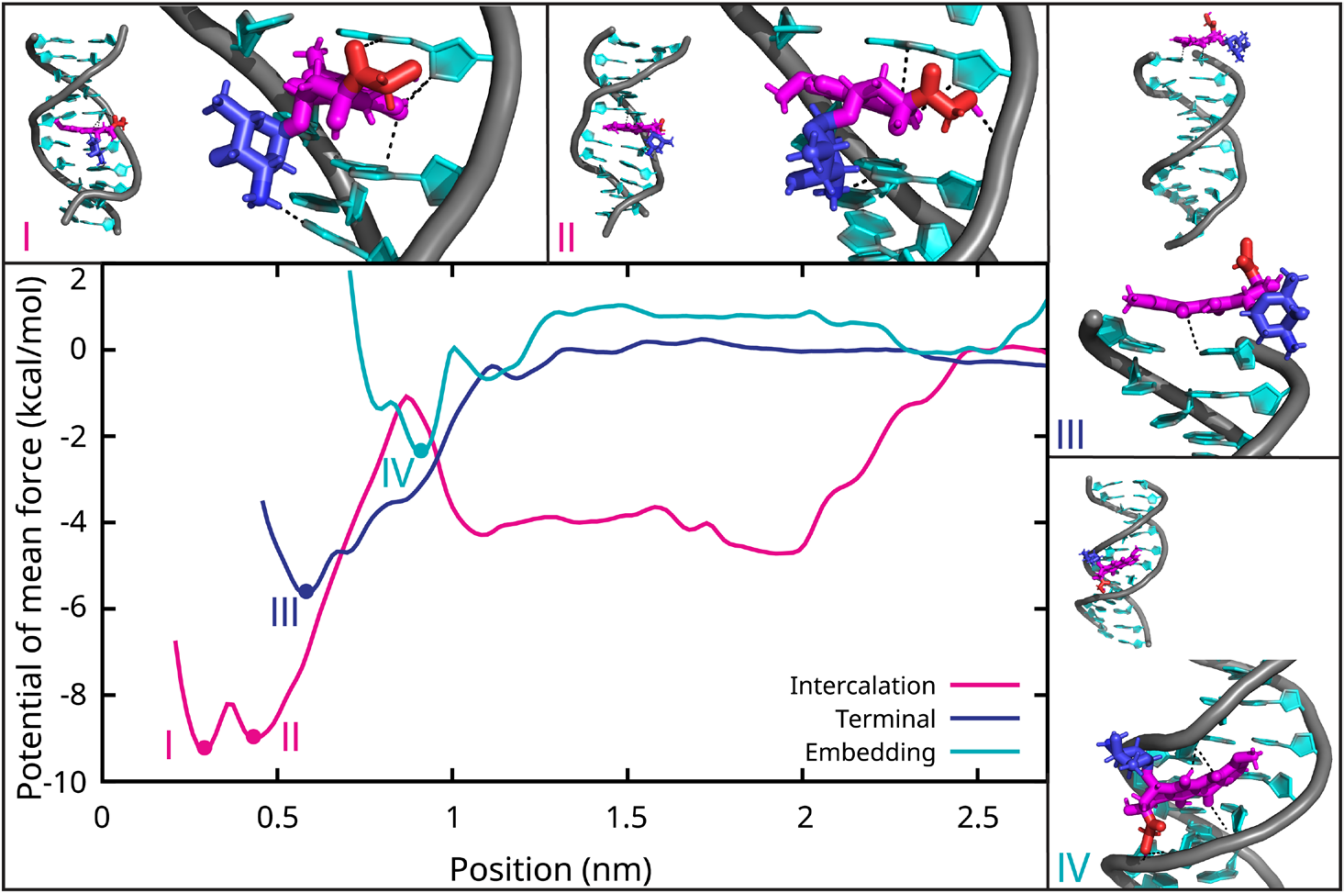
Potential of mean force curves for the intercalation process, stacking on terminal bases, and embedding within the minor groove of the DNA. The conformations corresponding to the energy minima are highlighted. The position is the distance between doxorubicin (DOX) center of mass and the center of masses of the interacting DNA base pairs (see the Methods section). The DOX molecule is shown in magenta/red/blue sticks, while the DNA in a cyan/gray cartoon. Water and ions are omitted for clarity.

To evaluate the thermodynamics of the interaction modes between DOX and DNA, we performed PMF calculations (Figure 7). The PMF results aligned with the unbiased simulations, confirming that embedding in the minor groove is a weak binding mode. In contrast, stacking on the terminal bases is energetically favorable, while intercalation, although the most energetically favorable, is kinetically hindered by the energy barrier required for base pair opening.

The simplest interaction between DOX and DNA is the stacking of DOX on the terminal bases, a barrierless process that results in a DOX affinity to DNA terminal bases of 5.7 kcal/mol relative to DOX in bulk water (Figure 7 and Table S5). This stacking mode on terminal bases was also observed in the PMF of the intercalation (at 2.0 nm in Figure 7), resulting in a similar stacking affinity within the error bars (Figure S8). While moving of DOX along the DNA backbone requires negligible energy, base pair opening (at 1.1 nm) involves overcoming an energy barrier of 3.2 kcal/mol. Full intercalation of DOX between DNA bases leads to two energy minima, with the anthraquinone methoxy group facing either the major (0.43 nm) or the minor groove (0.29 nm). The full intercalation affinity reaches 9.0 kcal/mol relative to bulk water, indicating the high stability of DOX already intercalated into the dsDNA. The weakest interaction mode is the embedding in the minor groove, with an affinity of just 2.6 kcal/mol, which explains the observed spontaneous detachments of DOX from the minor groove in both dsDNA (Figure S3) and NS simulations. The most favorable embedded position (0.9 nm) occurs when the minor groove is tightly locked around the DOX molecule.

## 4 Conclusions

In this study, we performed MD simulations to explore the interactions between DOX and various DNA models. DOX is known to damage DNA, with its intercalation between DNA bases being a proposed key mechanism. However, our simulations of DNA duplexes revealed that DOX tended to stack on the terminal bases. This stacking interaction was kinetically favored over spontaneous intercalation, which was not observed in the DNA duplex structure. Only in rare instances, DOX embedded into the DNA minor groove. This stacking preference was also observed in the G‐quadruplex (G4) structure, where the large tetrad provided by G4 could accommodate two DOX molecules simultaneously. Additional DOX molecules were seen stacking on top of this newly formed DOX platform. Most notably, we observed the spontaneous intercalation of DOX between DNA neighboring base pairs in nucleosomal DNA, which we rationalized by its distinctive shape and increased torsional stress.

The inherent flexibility and structural variations of nucleosomal DNA, including localized bending, torsional stress, and interactions with histone proteins, likely create a more favorable environment for DOX intercalation compared to the more rigid and uniform B‐DNA. This increased flexibility may enhance the accessibility of specific sites, making them more prone to intercalation, which could have important implications for the effectiveness of DOX as a chemotherapeutic agent. These regions of enhanced DNA flexibility were confirmed in our simulations by analyzing their helical parameters, differing significantly from simple dsDNA. The helical parameters were non‐uniform even in the simulations of bare nucleosomal DNA. The behavior of DOX in regions of increased torsional stress may have relevance beyond NS, potentially affecting other biological processes that create increased torsional stress, such as DNA replication and transcription.

Finally, we investigated the individual interaction modes of DOX using PMFs. These results indicated that while intercalation into the DNA duplex was energetically favorable, it was kinetically blocked by an energy barrier. This barrier may be lower in the case of bent DNA within the NS, which could explain the spontaneous intercalation observed in these regions. However, the PMFs suggest that if DOX intercalates between the DNA base pairs in the bent DNA structure, it will stay intercalated also when the torsional stress in the DNA chain is released. Our simulations provide novel insights into the intercalation process, highlighting the critical role of DNA tertiary and quaternary structures in modulating DOX interactions and offering potential avenues for optimizing DOX‐based therapies.

## Author Contributions

The manuscript was written through contributions of all authors. All authors have given approval to the final version of the manuscript.

## Conflicts of Interest

M.O. has a share in InSiliBio biosimulation company.

## Supporting information


**Data S1** Supporting Information.
**Tables S1–S4:** Overview of the performed simulations.
**Table S5:** Free energy barriers of DOX on dsDNA.
**Figure S1:** Starting positions of the DOX molecule in dsDNA simulations.
**Figure S2:** Binding of a DOX molecule to the DNA duplex.
**Figure S3:** Time evolution of the interaction of DOX with dsDNA.
**Figure S4:** Binding of a DOX molecule to the G‐quadruplex (G4).
**Figure S5:** Evolution of the rise of the selected NS sequence.
**Figure S6:** Evolution of the rise between NS base pairs.
**Figure S7:** Evolution of the twist values.
**Figure S8:** Potential of mean force of DOX on dsDNA.

## Data Availability

Datasets for all figures in the main text and Supplementary Information, along with representative structures and selected trajectories, is available at zenodo.org (DOI: 10.5281/zenodo.14501055).
