# Supplementary material for: Atomistic Insights Into Interaction of Doxorubicin With DNA: From Duplex to Nucleosome
Source: J Comput Chem. 2025 Jan 26;46(3):e70035. doi: 10.1002/jcc.70035 (PMC11771641; doi:10.1002/jcc.70035)
Supplement: Supplementary file 1 — Data S1 Supporting Information. Tables S1–S4: Overview of the performed simulations. Table S5: Free energy barriers of DOX on dsDNA. Figure S1: Starting positions of the DOX molecule in dsDNA simulations. Figure S2: Binding of a DOX molecule to the DNA duplex. Figure S3: Time evolution of the interaction of DOX with dsDNA. Figure S4: Binding of a DOX molecule to the G‐quadruplex (G4). Figure S5: Evolution of the rise of the selected NS sequence. Figure S6: Evolution of the rise between NS base pairs. Figure S7: Evolution of the twist values. Figure S8: Potential of mean force of DOX on dsDNA. [file JCC-46-0-s001.docx]

Supporting Information

for

# Atomistic Insights into Interaction of Doxorubicin with DNA: From Duplex to Nucleosome

Andrea Nedělníková^1^, Petr Stadlbauer^1,2^, Michal Otyepka^1,3^, Petra Kührová^1*^ and Markéta Paloncýová^1*^

^1^ Regional Center of Advanced Technologies and Materials, The Czech Advanced Technology and Research Institute (CATRIN), Palacký University Olomouc, Šlechtitelů 27, 779 00 Olomouc, Czech Republic

^2^ Institute of Biophysics of the Czech Academy of Sciences, Královopolská 135,

612 65 Brno, Czech Republic

^3^ IT4Innovations, VŠB – Technical University of Ostrava, 17. listopadu 2172/15, 708 00 Ostrava-Poruba, Czech Republic

* corresponding authors, e-mails: petra.kuhrova@upol.cz, marketa.paloncyova@upol.cz

***Table S1:*** *List of performed simulations of solvated biosystems without DOX molecules.*

| **System** | **Box size (nm)** | **Ions** | | **Length (μs)** |
| --- | --- | --- | --- | --- |
|  |  | **K^+^** | **Cl^-^** |  |
| Free dsDNA | 4.4 × 4.6 × 6.4 | 43 | 21 | 1 × 1 |
| Free G4 | 5.9 × 6.6 × 7.5 | 44 | 26 | 1 × 1 |
| Free NS | 15 × 15 × 15 | 495 | 289 | 1 × 1 |
| Free NS duplex | 6.5 × 7.0 × 7.8 | 37 | 20 | 1 × 0.1 |

***Table S2:*** *Overview of 30 dsDNA simulations with a DOX molecule. The simulation length indicates the minimum, if the DOX-dsDNA interaction did not occur at least 20 ns before the simulation end, the simulation was prolonged. The Results section shows the resulting binding mode (BM), number of its observations (#), mean time of the DOX binding (BT), and the frequency of observed fraying of the terminal base pairs.*

| **Simulation settings** | | | | **Results** | | | |
| --- | --- | --- | --- | --- | --- | --- | --- |
| **Box size**  **(nm)** | **Ions** | | **Length**  **(μs)** | **BM** | **#** | **BT^1^ (ns)** | **Fraying** |
|  | **K^+^** | **Cl^-^** |  |  |  |  |  |
| 7×7×7 | 52 | 31 | min. 0.1 | Stacking TBP C1:G24 | 15× | 85 | 3× |
| 7×7×7 | 52 | 31 | min. 0.1 | Stacking TBP G12:C13 | 12× | 53 | 3× |
| 7×7×7 | 52 | 31 | min. 0.8 | Minor groove | 3× | 27 | 1× |
| 7×7×7 | 52 | 31 | min. 0.1 | Intercalation | - | - | - |

^1^The mean binding time translates into the binding rate constant on the order of 10^9^ M^-1^s^-1^, i.e. the binding rate it essentially diffusion-limited.

***Table S3:*** *Overview of simulations of biosystems with multiple DOX molecules, simulation details and their number and lengths.*

| **System** | **Box size**  **(nm)** | **Ions** | | **Length**  **(μs)** |
| --- | --- | --- | --- | --- |
|  |  | **K^+^** | **Cl^-^** |  |
| **dsDNA + 3 DOX** | 9×9×10 | 94 | 75 | 3 × 1 |
| **G4 + 5 DOX** | 9×7×6 | 47 | 31 | 4 × 0.5 |
| **NS + 10 DOX** | 14×15×15 | 477 | 281 | 3 × 0.7 |

***Table S4:*** *List of performed simulations connected to the potential of mean force calculation, number of simulated windows/replicas and their length.*

| **System** | **Box size**  **(nm)** | **Ions** | | **Unbiased** | **Pulling** | **PMF** | |
| --- | --- | --- | --- | --- | --- | --- | --- |
|  |  |  |  | **Length**  **(ns)** | **Length**  **(ns)** | **Number of windows** | **Length**  **(ns)** |
|  |  | **K^+^** | **Cl^-^** |  |  |  |  |
| **Intercalation** | 5.6 × 6.6 × 6.4 | 37 | 20 | 1 × 100 | 23; 27 | 59 | 50 |
| **Embedding** | 6.9 × 7.0 × 5.9 | 46 | 23 | 1 × 100 | 20; 28 | 53 | 50 |
| **Terminal Bases** | 6.3 × 4.5 × 8.1 | 27 | 10 | 1 × 100 | 15; 29 | 58 | 50 |


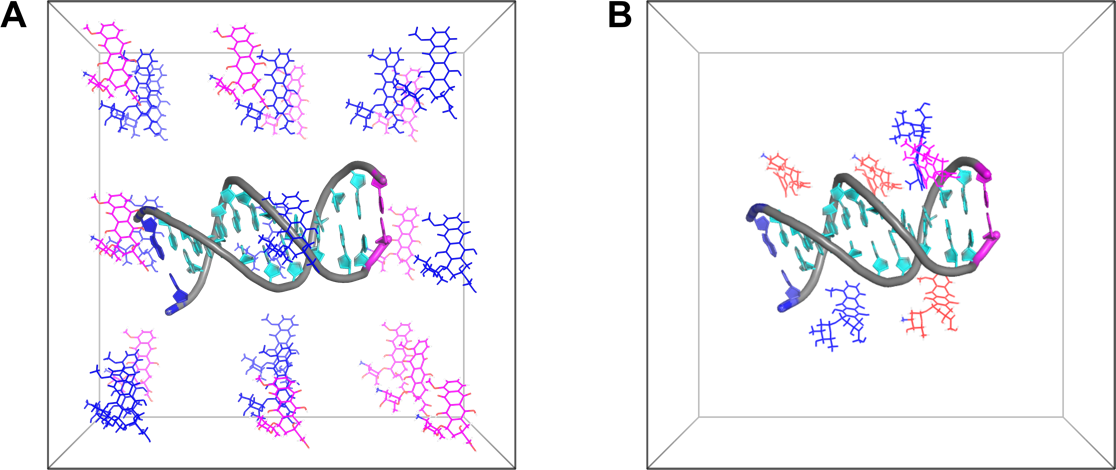


***Figure S1:*** *Starting positions of DOX molecule in dsDNA simulations. In 24 models (A), DOX molecules were positioned equidistantly along the box edges, except near the terminal base region. In 6 models (B), DOX was oriented differently and placed near the DNA duplex. At the end of the simulations, molecules marked in blue were stacked on terminal base pair C1:G24 (15 models), while those in magenta stacked on terminal base pair G12:C13 (12 models). Molecules marked in red interacted with the minor groove (3 models).*





***Figure S2:*** *Binding of a DOX molecule to the DNA duplex. A) DOX molecule bound to the terminal base pair C1:G24 of dsDNA. The DOX molecule is oriented with its sugar moiety toward the terminal base pair, positioned in the major groove, with preferred interactions at the 3’-end of the DNA. The graph shows the orientation of DOX during the simulation when bound. The position of the geometric center of anthraquinone ring C projected into the base pair plane is represented by the probability density graph on the right, while the orientation of the anthraquinone moiety (from ring C to ring A) is shown by a vector. The vector size reflects population density and direction preference. Red vectors indicate the daunosamine moiety pointing towards the base pair, while blue vectors indicate the direction away from the base pair. For clarity, only the positions of the C(N1) and G(N9) atoms of the bases are shown. Top and side view of the representative conformer, highlighting key parts of the DOX-dsDNA complex, is on the left: anthraquinone moiety in violet, daunosamine in blue, anchor in red, stem guanines in bluish tones, other nucleotides in cyan, the backbone in dark grey, and channel cations in white.* *B) DOX molecule bound to the terminal base pair G12:C13 of dsDNA. The DOX molecule is oriented with its sugar moiety toward the terminal base pair, this time in the minor groove, with preferred interactions at the 5’-end of the DNA. C) DOX molecule oriented by its anchor toward the terminal base pairs, without a preferential orientation toward specific terminal bases. D) DOX intercalated into nucleosome, showing limited rotation freedom. Daunosamine is aiming towards the C2:G3 base pair. The average plane of the two neighbouring base pairs was used for the projection.*


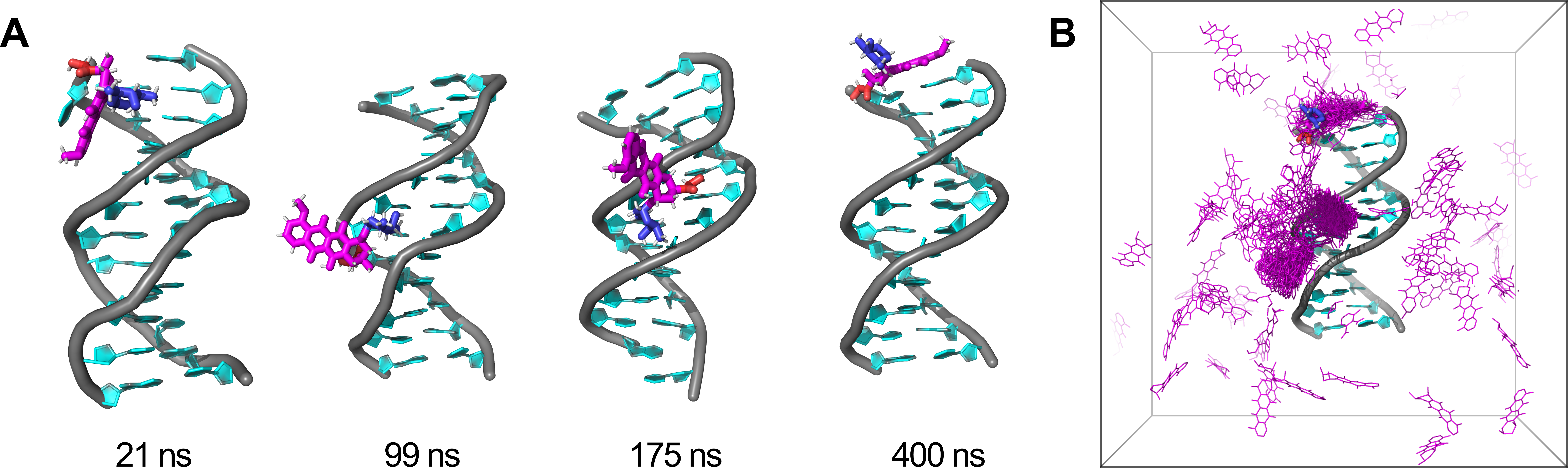


***Figure S3:*** *Time evolution of the interaction of DOX with dsDNA. A) Snapshots illustrating the interaction of DOX with the dsDNA. The first contact between DOX and dsDNA occurred at 21 ns, but DOX was repelled into the solvent before re-engaging at 99 ns, with the anchor and daunosamine moieties oriented toward the minor groove. DOX then moved along the minor groove before being released into the solvent again. Around 340 ns, DOX began interacting with the terminal bases, maintaining a stable interaction until the end of the simulation. B) Localization of the anthracycline rings during the simulation, superimposed on a single dsDNA structure. The DOX anthraquinone moiety is shown in magenta, the anchor in red, and the daunosamine moiety in blue. The dsDNA is depicted as a grey-cyan cartoon, with water and ions omitted for clarity.*





***Figure S4:*** *Binding of a DOX molecule to the G-quadruplex (G4). A) Two DOX molecules bound to the 5’-tetrad. B) Two DOX molecules bound to the 5’-tetrad in a different way. C) One DOX molecule bound to the 3’-tetrad. D) One DOX molecule bound to the 5’-tetrad. See the legend to Figure S2 or main text Figure 3 for graphics explanation.*


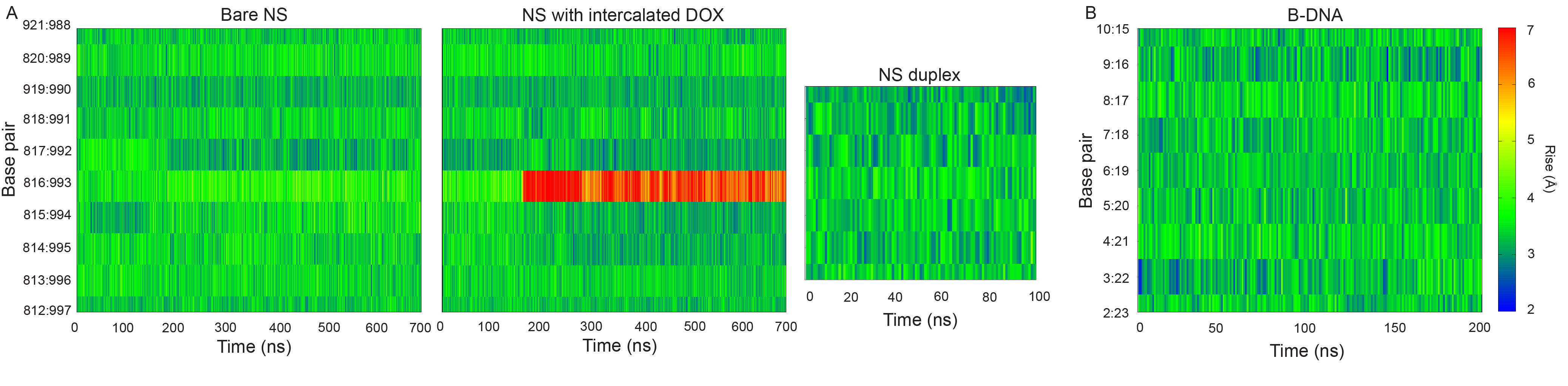


***Figure S5:*** *A) Evolution of the rise between base pairs in 812:997-921:988 region during simulations of bare NS, NS with intercalated DOX, and the NS duplex. The graph for the NS duplex is overlaid with simulations of NS for direct comparison. B) Evolution of the rise between base pairs in dsDNA (B-DNA) simulation. In the NS duplex and B-DNA simulations, terminal base pairs were excluded from the visualization due to base pair fraying.*


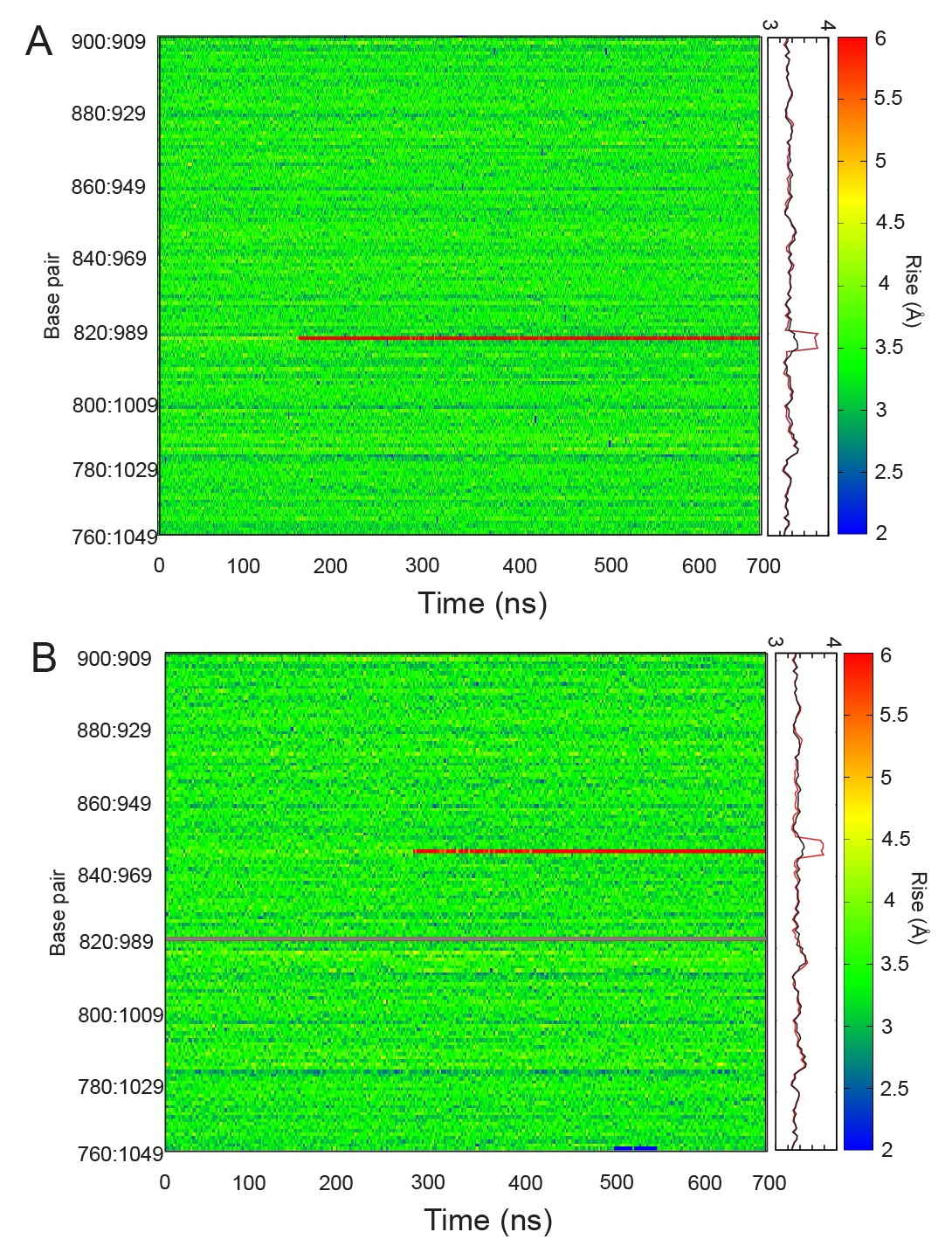


***Figure S6:*** *A)+B) Evolution of rise between NS base pairs during two independent simulations, along with its running average over 5 base pair steps calculated using sliding window (red curve). For comparison, the running average of rise parameter from the simulation of bare NS without DOX is shown (black curve). The grey line represents data excluded because of an artificial high-rise value calculated by cpptraj. The program was unable to calculate the rise parameter for base pair 820:989 due to the loss of base pairing.*


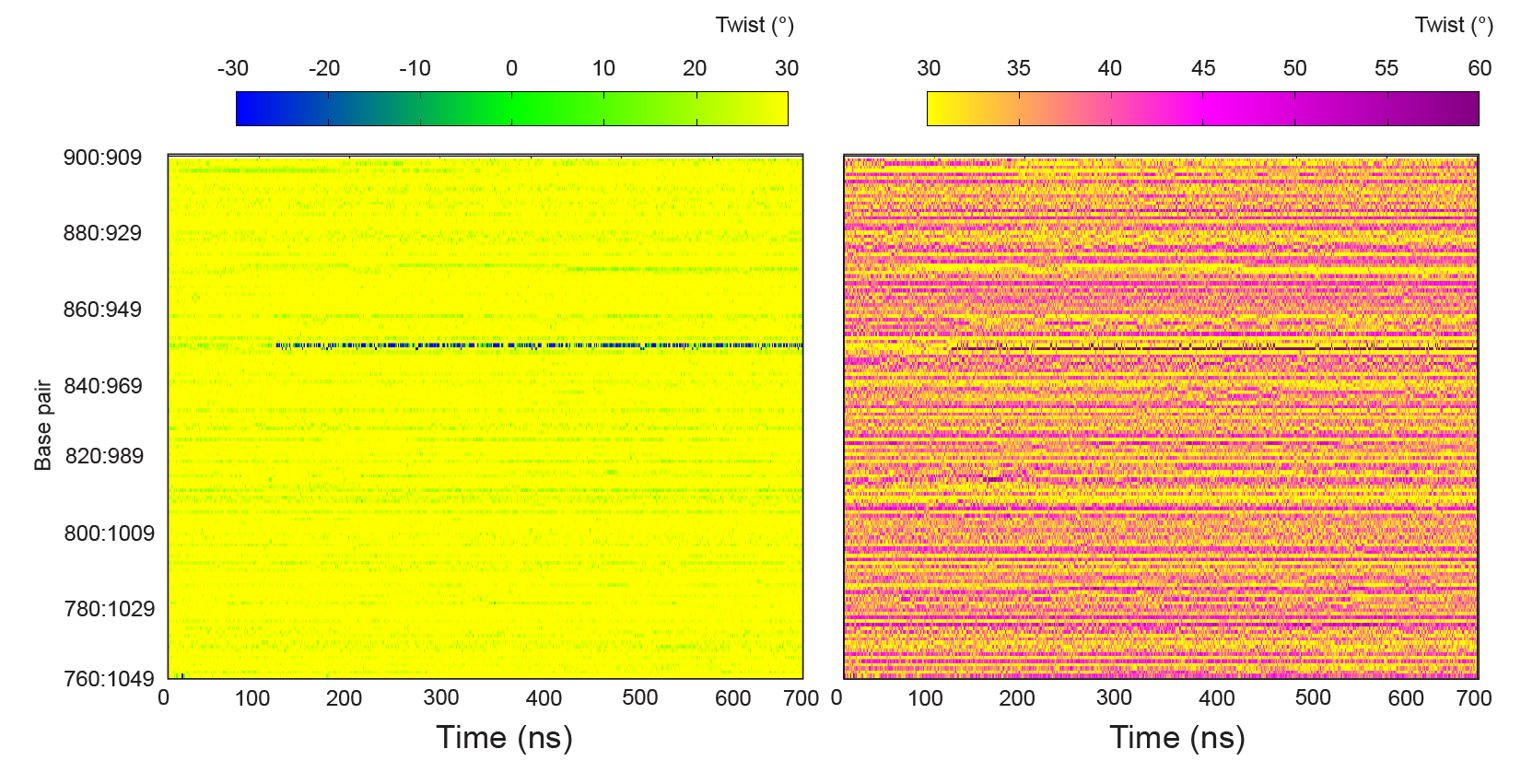


***Figure S7:*** *Nucleosome simulation results comparing regions of low and high twist values, using two different scales. The left graph highlights low twist values, from -30 to 30 degrees, while the right graph focuses on higher twist values, from 30 to 60 degrees. Both graphs show the same simulation as discussed in the main text (Figure 6).*


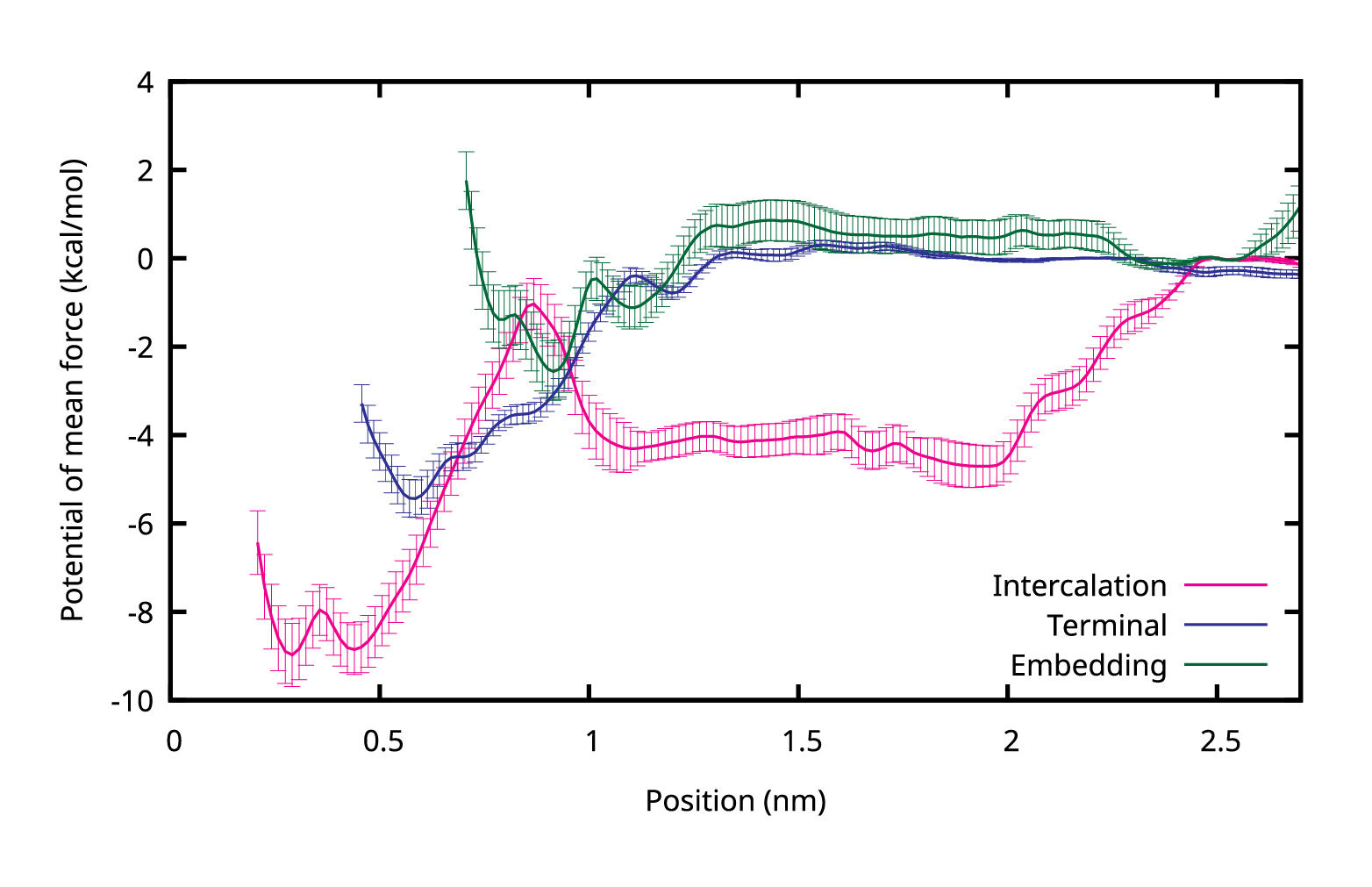


***Figure S8:*** *Potential of mean force (PMF) of individual binding modes of DOX with dsDNA including standard deviations.*

*Table S5: Binding energies of individual binding modes together with the height of the intercalation energy barrier.*

| **Binding mode** | **Binding energy (kcal/mol)** | **Energy barrier (kcal/mol)** |
| --- | --- | --- |
| **Intercalation** | -9.0 | 3.2 |
| **Terminal** | -5.7 | *barrierless* |
| **Embedding** | -2.6 | *barrierless* |
